# Structural diversity and unity amongst axonemal dynein assembly factors

**DOI:** 10.1242/jcs.264247

**Published:** 2025-10-28

**Authors:** Muyang Ren, Siham A. Jaleel, Stephen M. King, Girish R. Mali

**Affiliations:** ^1^ Sir William Dunn School of Pathology, University of Oxford, South Parks Road, Oxford, OX1 3RE, UK; ^2^ Department of Molecular Biology and Biophysics, University of Connecticut Health Center, 263 Farmington Avenue, Farmington, CT 06030-3305, USA; ^3^ School of Biochemistry, University of Bristol, University Walk, Bristol, BS8 1TD, UK

**Keywords:** Cilia, Axonemal dyneins, Molecular motors, Assembly factors, Protein folding

## Abstract

The essential beating motion of cilia is powered by large multi-subunit complexes called axonemal dynein arms, which are synthesised through a dedicated assembly pathway. Dynein arm assembly is shepherded by nineteen axonemal dynein assembly factors (DNAAFs), which operate in an intricate chaperone relay to promote the protein folding and assembly of individual dynein arm subunits into functional motor complexes in the cytoplasm, followed by their transport into motile cilia. Genetic variants that block the assembly pathway underlie cilia-related human pathologies, including the multi-organ motile ciliopathy called primary ciliary dyskinesia. This structure-focused Perspective summarises recent progress on the DNAAFs and spotlights their emerging roles as important players in the biology of motile cilia.

## Teaser

**Summary:** Multi-subunit motors known as axonemal dynein arms power ciliary beating in eukaryotes. This Perspective spotlights the structural diversity of the axonemal dynein assembly factors (DNAAFs), which assist dynein arm synthesis.

## Introduction

Motile cilia are microtubule-based cellular extensions found across a wide breadth of eukaryotes. They perform diverse motility- and cell signalling-related functions, from locomotion in single-celled protozoa to removing pathogens from the respiratory tracts of mammals. The central scaffold of motile cilia, termed the axoneme, comprises nine outer doublet microtubules surrounding a central pair of singlet microtubules. ATP-powered multi-subunit motor protein complexes, called the axonemal outer and inner dynein arms (ODAs and IDAs, respectively), attach to the outer doublet microtubules along most of the length of the axonemes to power ciliary oscillations.

Genetic perturbations that abrogate dynein arm functions or disrupt their synthesis result in a severe multi-organ motile ciliopathy called primary ciliary dyskinesia (PCD), which affects ∼1 in 7500 newborns per year. PCD is characterised by severe lung complications and left–right body axis defects and can involve male and female infertility (Wallmeier et al., 2020). More recent studies have linked ciliary motility defects to congenital heart disease, epilepsy, age-related ventriculomegaly, neuropsychiatric disorders and multiple sclerosis (Klena et al., 2017; Faubel et al., 2022; Eisenhuth et al., 2025; Alhassen et al., 2021; Bigotte et al., 2025 preprint).

**Fig. 1.**
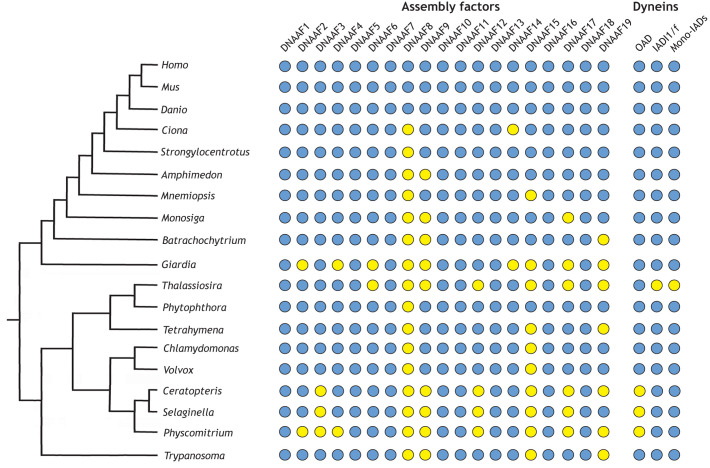
**Presence of assembly factors in extant eukaryotes.** This diagram illustrates the occurrence of DNAAFs in a broad array of ciliated eukaryotes based on BLAST searches using the human orthologue as the query. BLAST searches were done at NCBI (https://blast.ncbi.nlm.nih.gov/) using the default parameters and the non-redundant database restricted to each indicated organism. Organisms are grouped based on the recently recovered, robustly rooted eukaryotic tree of life (Williamson et al., 2025). Blue indicates that a clear orthologue is present; yellow indicates that no orthologue has been reliably identified. The three columns on the far right indicate the presence or absence of outer arm, inner arm I1/f and/or monomeric inner arm dynein heavy chains in these organisms (denoted OAD, IADI1/f and mono-IADs, respectively). This Perspective uses the consensus nomenclature as defined by Braschi et al. (2022).

Dynein arms are megadalton-scale multi-protein complexes each comprising 15–20 precisely arranged individual subunits. All subunits are pre-assembled in the cytoplasm during ciliogenesis. Thousands of pre-assembled dynein arms are then packaged and undergo regulated deployment into growing cilia to power beating. Dynein arms are transported into cilia by a conserved bidirectional transit system called intraflagellar transport (IFT). Cytoplasmic pre-assembly and ciliary import via IFT are orchestrated by a set of nineteen proteins known as axonemal dynein assembly factors (DNAAFs), many of which were first discovered over 20 years ago in the unicellular ciliate *Chlamydomonas reinhardtii* (Braschi et al., 2022; Fowkes and Mitchell, 1998). Soon after, clinical observations and genetic analysis in human cases with motile cilia defects, combined with functional analysis in a Medaka (rice fish) mutant, offered the first link between DNAAFs and the human motile ciliopathy PCD (Omran et al., 2008). Nineteen vertebrate DNAAFs have been characterised so far. Seven DNAAFs are conserved across all extant eukaryotic lineages that build motile cilia, including *Plasmodium* species, which assemble their flagella inside the cytoplasm. One DNAAF appears to have evolved more recently and might represent a vertebrate-specific specialisation of the dynein arm assembly process. The remaining DNAAFs show a variable degree of conservation across the eukaryotic tree of life (Fig. 1; Tables S1, S2).

Numerous functional studies using different model organisms (mice, zebrafish, Medaka, *Xenopus*, *Drosophila*, *Tetrahymena* and *Chlamydomonas*), combined with genetic data from individuals with PCD, highlight the unique and non-redundant functions of each DNAAF protein. At a cellular level, DNAAFs are proposed to function within poorly defined subcellular compartments, variously termed dynein axonemal particles (DynAPs), kl-granules, dynein assembly factories and R2HADs (Huizar et al., 2018; Fingerhut and Yamashita, 2020; King, 2021). These dynein assembly foci are thought to contain both protein and RNA, suggesting their role as ribonucleoprotein (RNP) granules related to axonemal dynein arm biosynthesis. This topic is covered in another Perspective in this issue (Wallingford et al., 2025).

**Fig. 2.**
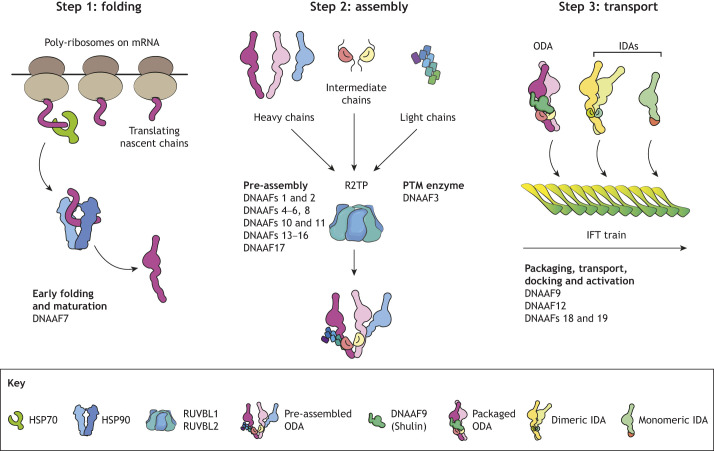
**Dynein assembly pathway and putative roles of DNAAFs that work within it.** Axonemal dynein arm motor subunits (heavy chains, intermediate chains and light chains) are assembled in a multi-step dynein assembly pathway involving steps of folding, assembly and transport. DNAAFs are thought to operate at distinct steps. HSP70 proteins likely engage nascent dynein heavy chain, intermediate chain and light chain subunits as they emerge from the ribosome exit tunnel. Specific DNAAFs then likely hand over semi-folded subunits to the HSP90 chaperone and its co-chaperone R2TP complex for further folding and assembly. Many DNAAFs are proposed to function at this step with RUVBL1/2. Pre-assembled ODA motors are finally packaged into an inhibited closed conformation by DNAAF9 (Shulin) for stable attachment to IFT trains for ciliary import. IDAs might undergo similar packaging for IFT transport via currently unknown factors. Genetic variants that block any step of assembly can cause severe motile ciliopathies such as PCD. PTM, post-translational modification.

At a molecular level, several DNAAFs are proposed to cooperate with housekeeping molecular chaperones of the HSP70 and HSP90 families for initial folding and maturation, and with the co-chaperone ATPases RUVBL1 and RUVBL2 (collectively RUVBL1/2), which form a platform of hetero-hexameric rings with two other cofactors (Fig. 2). Altogether, this is referred to as the R2TP complex, which executes dynein arm pre-assembly (Dong et al., 2014; Hartill et al., 2018; Li et al., 2017; Mali et al., 2018; Omran et al., 2008; Tarkar et al., 2013; Zhao et al., 2013; Zur Lage et al., 2018). A few DNAAFs also bind IFT proteins to couple pre-assembled dynein arms with polymeric train-like IFT assemblies for ciliary import (Ahmed et al., 2008; Issa et al., 2025; Mali et al., 2021; Taschner et al., 2017) (Fig. 2). Although their molecular mechanisms are beginning to emerge, a structural understanding of the DNAAFs as a diverse group of proteins working within the dynein assembly pathway is crucially lacking. This Perspective aims to spotlight the structural diversity and unifying features of the DNAAFs, with a view to posing testable hypotheses on their potential molecular functions in the cell for future investigation.

## Bringing order within disorder at a large scale

In contrast to cytoplasmic dynein-1 and -2, axonemal dynein motors undergo a distinct, DNAAF-shepherded biosynthetic programme of assembly. The need for multiple chaperone systems and dedicated assembly factors underscores the scale of this biosynthetic operation. An average cilium with a length of 10 μm requires an estimated 10,000 individual ODAs and IDAs for beating. Coordinating the folding, assembly and transport of several thousand dynein arms into a single cilium is already a significant challenge for a cell. This challenge becomes even greater in multiciliated cells, which must exquisitely scale their biosynthetic capacity to produce ∼2 TDa of protein content (King, 2021). In vertebrates, dynein arm assembly is intricately linked to the biogenesis of hundreds of motile cilia. Dynein arms are thought to be deployed into growing cilia in a highly regulated manner as multiciliated cells differentiate and mature, adding another level of complexity to the task. Coordinating protein folding and assembly whilst preventing aggregation and then timing the transport of pre-assembled arms into cilia whilst keeping their motor activities switched off is a sizeable logistical challenge. This challenge is met by the use of a highly efficient chaperone relay in the cell (Mali et al., 2018).

Dynein arm assembly involves a multi-step biosynthetic pathway, where most DNAAFs operate during the cytoplasmic folding and assembly phases. Some DNAAFs bind pre-assembled axonemal dynein arms to promote ciliary import, final delivery and docking (Bonnefoy et al., 2024; Dai et al., 2018; Desai et al., 2015; Mali et al., 2021). DNAAFs are not incorporated into the final fully functional motor holocomplexes that dock onto the axonemal microtubules. Therefore, they are not regulators of dynein arm motor activity per se. Their main function in the cell appears to be to shield intermediate folding states from off-target interactions, stabilise partially assembled intermediates or aid in ciliary import. All these roles require DNAAFs to make highly transient interactions.

**Fig. 3.**
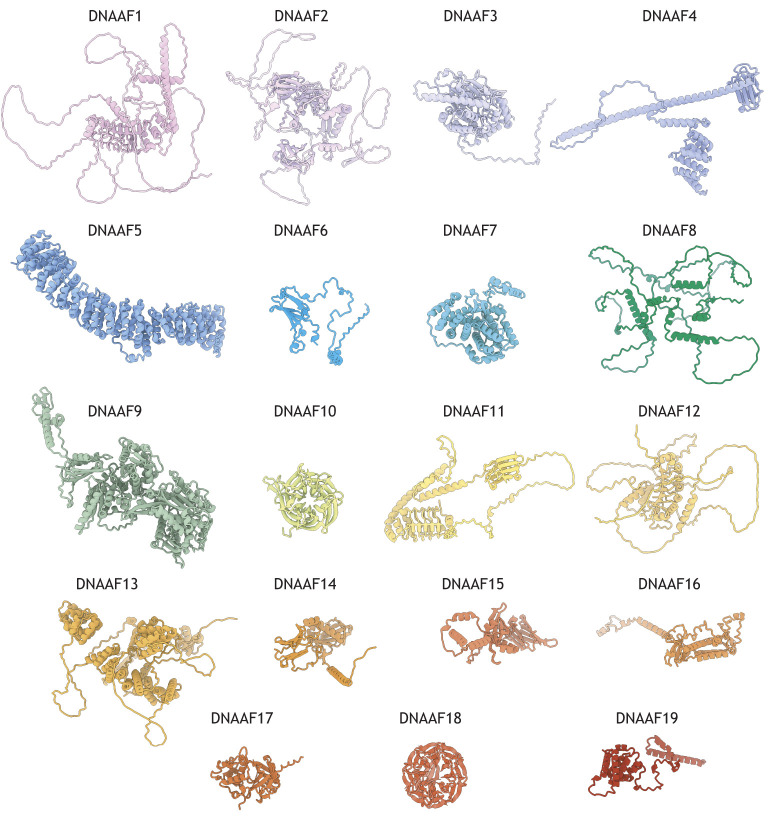
**AlphaFold predictions highlight the structural diversity amongst DNAAF proteins.** Predicted structures of the nineteen human DNAAFs were obtained from the AlphaFold EBI database (https://alphafold.ebi.ac.uk/; data used under the terms of a CC-BY 4.0 licence). Structural models were visualised using ChimeraX (Goddard et al., 2018; https://www.cgl.ucsf.edu/chimerax/).

Structural predictions based on AlphaFold3 modelling show that intrinsic disorder is a common feature of several DNAAFs, further underscoring the transient and labile nature of their molecular associations (Fig. 3) (Abramson et al., 2024). Intrinsically disordered regions could permit transient, multivalent interactions required for recruiting multiple chaperones or bridging subunit assembly. Additionally, these regions could undergo a disorder-to-order transition upon binding to their cognate region in a partner protein (Latysheva et al., 2015). The topic of inherent disorder in DNAAFs has been covered elsewhere (King, 2024). Interspersed between long stretches of intrinsically disordered regions, the DNAAFs also contain well-defined domains. Ordered domains could harbour sites of more stable protein–protein interactions – for instance, between DNAAFs or between DNAAFs and their dynein client subunits – and at least one DNAAF has been found to exhibit enzymatic activity within an ordered domain.

## Clustering of DNAAFs by protein domains highlights potential molecular roles

Several DNAAFs share common protein domains. Based on these, DNAAFs can be broadly categorised into six groups, including those with either a leucine-rich repeat (LRR) domain, a protein interacting with HSP90 (PIH) domain, a WD domain (comprising repeating units ending with tryptophan–aspartic acid), or tetratricopeptide repeats (TPRs) with an RNA polymerase II-associated protein 3-like C-terminal (RPAP3_C, hereafter RPAP) domain. Some DNAAFs also contain a bipartite cysteine- and histidine-rich domain (CHORD)–SGT1 domain (CS domain). Three DNAAFs contain unique functional domains, and four have domains of unknown function (DUFs) (Table 1).

**Table 1.** Clustering of DNAAFs based on shared domains

Category	Name	Domains	Protein length (*n* amino acids)	Alias
LRR proteins	DNAAF1	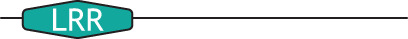	725	ODA7, LRRC50
	DNAAF11	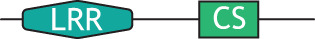	466	LRRC6
	DNAAF12	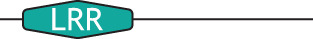	542	LRRC56
PIH domain proteins	DNAAF2	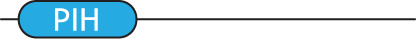	837	KTU, kintoun
	DNAAF6	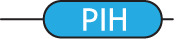	214	PIH1D3
	DNAAF14	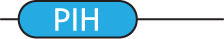	290	PIH1D1
	DNAAF15	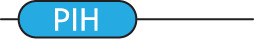	315	PIH1D2
WD repeat proteins	DNAAF10	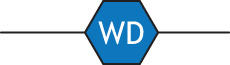	357	WDR92, Monad
	DNAAF18	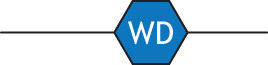	415	DAW1, WDR69, ODA16
TPR and RPAP domain proteins	DNAAF4	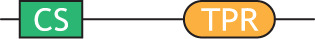	420	DYX1C1
	DNAAF13	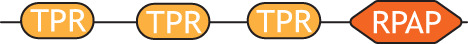	926	SPAG1
	DNAAF19	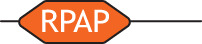	242	CCDC103
Proteins with unique domains	DNAAF5	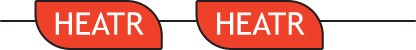	855	HEATR2
	DNAAF7	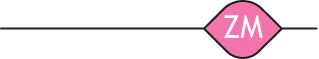	440	ZMYND10
	DNAAF9	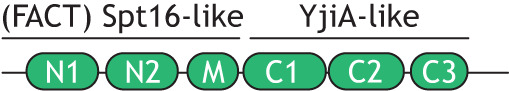	1177	C20ORF194, Shulin
Proteins with domains of unknown function	DNAAF17	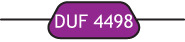	267	C11ORF70, CFAP300
	DNAAF3	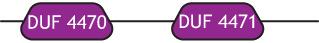	541	C19ORF51
	DNAAF8	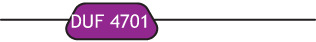	520	C16ORF71
	DNAAF16	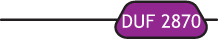	290	C21ORF59, CFAP298

Protein domain architecture for each DNAAF was obtained using SMART web tool (http://smart.embl-heidelberg.de/) and UniProt (https://www.uniprot.org/). See Braschi et al. (2022) for other aliases. HEATR, HEAT repeat domain; M, middle domain; ZM, zinc finger MYND domain.

## LRR proteins

DNAAF1 (also known as ODA7 and LRRC50), DNAAF11 (also known as LRRC6) and DNAAF12 (also known as LRRC56) contain LRR domains, which are 22–28-amino-acid motifs commonly found in proteins with diverse functions and cellular locations. LRR repeats form non-globular, crescent-like structures associated with protein–protein interaction interfaces (Fig. 3). The concave surfaces consist of parallel β-strands, and the convex face is composed of more diverse secondary structures, including helices (Ng and Xavier, 2011). Although none of these DNAAFs have experimentally determined structures, AlphaFold3 modelling places their LRR repeats amongst regions with varying degrees of disorder.

DNAAF1 has been well studied in *Chlamydomonas* and zebrafish, where its loss impacts dynein heavy chain stability (Freshour et al., 2007; Mitchison et al., 2012). DNAAF11 contains a p23 crystallin-like domain commonly found in HSP90 co-chaperones and might participate with DNAAF7 (also known as ZMYND10) in a chaperone relay to advance the HSP90 cycle during dynein heavy chain folding and maturation (Mali et al., 2018). DNAAF12 was initially defined as part of a complex with the docking-complex subunits needed for ODA maturation (Bonnefoy et al., 2018; Desai et al., 2015). Recent work has revealed that knock-out of DNAAF12 in *Trypanosoma brucei* affects the presence of the distal docking complex. DNAAF12 associates with some, but not all, IFT trains and might be involved in the proper assembly of the distal dynein docking complex. Whether DNAAF12 has a direct connection to ODA transport and how its loss impacts ODA distribution in cilia remain unclear (Bonnefoy et al., 2024).

## PIH proteins

The PIH domain was first discovered in budding yeast as part of an HSP90 interactor named Pih1 (Zhao et al., 2005). The human proteome contains four PIH proteins – DNAAF2 (also known as KTU), DNAAF6 (also known as PIH1D3), DNAAF14 (also known as PIH1D1) and DNAAF15 (also known as PIH1D2) – which are orthologous to the yeast Pih1 protein, a component of the R2TP co-chaperone complex. In vertebrates, PIH proteins are proposed to assemble distinct sets of axonemal dynein arm subtypes (Yamamoto et al., 2010). Notably, DNAAF14 and DNAAF15 have not yet been linked to human motile ciliopathy or PCD cases. This is likely due to their vital roles in assembling other essential cellular machinery, such as RNA polymerases, in addition to axonemal dynein arms, indicating that their genetic functional loss might be embryonically lethal (Kakihara and Houry, 2012). *DNAAF2* and *DNAAF6* are classical PCD genes, with *DNAAF6* being X-linked (Paff et al., 2017). Currently, there are no experimental structures available for these PIH proteins.

## WD proteins

DNAAF10 (also known as Monad and WDR92) and DNAAF18 (also known as DAW1, WDR69 and ODA16) contain highly conserved WD40-repeat domains (Patel-King et al., 2019; Taschner et al., 2017). The WD domain forms a toroidal β-propeller structure characterised by repeating units of ∼40–60 amino acids that often end with a tryptophan–aspartic acid dipeptide motif. DNAAF10 is thought to bridge the R2TP complex with the Prefoldin complex – two major chaperoning systems that cooperate in the cytoplasm for dynein arm assembly (Zur Lage et al., 2018). DNAAF10 is predicted to have seven WD repeats, each forming a four-stranded β-sheet arranged into a β-propeller with a single large protrusion derived from two extended loops. DNAAF18 is involved in ODA transport in *Chlamydomonas* flagella as an IFT adaptor via interaction with IFT46 (Taschner et al., 2017). Human DNAAF18 has an eight-bladed β-propeller structure, as determined using X-ray crystallography. However, its role in ODA transport remains unclear due to a lack of biochemical interaction with human IFT46, unlike its *Chlamydomonas* orthologue (Wang et al., 2020).

## TPR and RPAP proteins

DNAAF4 and DNAAF13 (also known as SPAG1) both feature TPRs, which are 34-amino-acid motifs that repetitively form helix-turn-helix structures. TPR domain proteins typically interact with C-terminal peptide motifs ending in EEVD, as found in HSP70 and HSP90 proteins (Scheufler et al., 2000). These domains are thought to form flexible scaffolds that facilitate protein–protein interactions for the assembly of multi-protein complexes (Martino et al., 2018). DNAAF4 also contains a CS domain (similar to DNAAF11), possibly aiding heat-shock protein chaperone binding (Tarkar et al., 2013). In addition, DNAAF13 and DNAAF19 (also known as CCDC103) share an RPAP domain, making them paralogues of the human RPAP3 protein, a canonical R2TP complex subunit involved in RNA polymerase assembly (Boulon et al., 2010). This implicates DNAAF13 and DNAAF19 as putative components of non-canonical R2TP-like complexes [for example, RUVBL1/2–SPAG1–DNAAF2 (R2SD) or RUVBL1/2–SPAG1–PIH1D2/PIH1D3 (R2SP) complexes] during dynein arm assembly. Structural data for these DNAAFs is limited. The first and third TPR domains and the RUVBL1/2-binding domain of DNAAF13 have been resolved using nuclear magnetic resonance and cryo-electron microscopy (cryo-EM), respectively (Chagot et al., 2019; Dermouche et al., 2021; Santo et al., 2025 preprint). However, the overall molecular architecture, especially when bound to RUVBL1/2, chaperones and potential client proteins, remains unresolved.

## DNAAFs with unique domains and DUFs

DNAAF5 (also known as HEATR2), DNAAF7 and DNAAF9 contain unique domains suggestive of distinct functions. DNAAF5 is predicted to contain ten HEAT repeats comprising tandem sequences of 37–47 amino acids that form α-helical structures, linked by a short loop. Overall, these tandem sequences linked by loops form a solenoid-like flexible structure to bridge protein–protein interactions (Diggle et al., 2014; Horani et al., 2018). DNAAF5 is structurally similar to TTI1 (TELO2-interacting protein 1), which cooperates with the R2TP complex in assembling phosphatidylinositol 3-kinase-like kinases (PIKKs), including mTORC1 (Pal et al., 2021). It might perform an analogous function for dynein arm pre-assembly as an accessory to R2TP and R2TP-like complexes.

DNAAF7 contains a C-terminal zinc finger myeloid, nervy and DEAF-1 (MYND) domain and is proposed to function at one of the earliest steps – dynein heavy chain folding (Mali et al., 2018). Owing to its zinc finger motif, commonly found in transcriptional co-regulators, DNAAF7 might couple nuclear transcriptional co-regulation with cytoplasmic dynein arm pre-assembly, potentially through interaction with DNAAF11 (Zariwala et al., 2013). DNAAF9 (known as Shulin in *Tetrahymena*) is a newly defined ODA packaging and inhibitory factor (Mali et al., 2021). It is a hybrid protein comprising an N-terminal segment (N1, N2 and M domains) that has an aminopeptidase fold highly similar to the N-terminal part of the Spt16 subunit of the FACT histone chaperone and a C-terminal segment that is similar to the bacterial GTPase YjiA and includes a stabilising nucleotide spanning the C1 and C2 domains. Shulin contains a C-terminal helical extension (C3 finger) that contacts the AAA1 site of the ODA γ-heavy chain. The cryo-EM structure of Shulin has revealed how it engages both its N- and C-terminal domains to bind ODAs, bridging the motor and tail domains of the motor complex to stabilise an inhibited conformation.

DNAAF3, DNAAF8, DNAAF16 (also known as CFAP298 and C21ORF59) and DNAAF17 (also known as CFAP300 and C11ORF70) contain different DUFs. Recently, DNAAF3 has been identified as a structural orthologue of *S*-adenosylmethionine-dependent arginine methyltransferases (Sakato-Antoku et al., 2024). It is involved in methylating axonemal dynein heavy chain subunits and is currently the only known assembly factor with enzymatic activity. DNAAF8 is largely disordered, with few known molecular interactions, but localises to DynAPs (Lee et al., 2020). A recent X-ray crystal structure of DNAAF16 has revealed a bilobed core containing two globular domains linked by a central α-helical bundle. One of the globular domains has a ubiquitin-like fold, making it similar to type II ubiquitin-like proteins (Yamamoto et al., 2025). DNAAF17, a small protein with a predicted globular fold, was first implicated in IFT-linked ODA ciliary distribution due to its IFT-like localisation in *Paramecium* cilia. However, it was later shown to co-immunoprecipitate with DNAAF2, indicating a direct interaction and suggesting a primary role in pre-assembly rather than in transport (Fassad et al., 2018; Höben et al., 2018). The molecular interactions formed by these DNAAFs remain poorly defined. Even so, these DUF-containing DNAAFs perform essential, yet mostly unexplored, functions related to axonemal dynein arm assembly. This is supported by the strong genetic association between PCD and variants of DNAAF3, DNAAF16 or DNAAF17 (Mitchison et al., 2012; Yamamoto et al., 2025; Fassad et al., 2018).

## Contextualising the roles of DNAAFs in dynein arm assembly

Domain-guided categorisation enables a rational placement of DNAAFs at distinct functional steps within the dynein assembly pathway. Cytoplasmic pre-assembly of dynein arms starts with the translation and folding of individual dynein arm subunit polypeptides. It is currently unclear whether the association and/or assembly of axonemal dynein heavy chain, intermediate chain and light chain, as well as other accessory subunits, occurs co-translationally, post-translationally or as a mix of both. DNAAF7 is needed for dynein heavy chain stability in mice. Without this protein, heavy chains are detected in cytoplasmic aggregates. It has therefore been proposed that DNAAF7 functions at one of the earliest steps: stabilising the co-translational folding of dynein heavy chains (Mali et al., 2018). It has also been suggested that DNAAF7 stabilises dynein intermediate chains (Cho et al., 2018). As it binds FKBP8 (an HSP90 co-chaperone) and DNAAF11 (a DNAAF that contains an HSP90-linked p23 domain) mutually exclusively, DNAAF7 has been proposed to be involved in advancing the HSP90 chaperone cycle to promote dynein heavy chain folding and maturation (Mali et al., 2018). DNAAF1 has also been implicated in heavy chain stability and might work alongside DNAAF7 at an early step (Mitchison et al., 2012). However, it has also been reported to associate with RUVBL1/2 and IFT88 and could therefore be involved in coupling dynein arm assembly with transport (Hartill et al., 2018).

Co-translational folding of individual dynein arm subunits likely involves HSP70 and HSP90 chaperones and is closely coupled to their R2TP–Prefoldin-assisted assembly into functional holocomplexes. All TPR, CS, PIH and RPAP domain-containing DNAAFs (DNAAFs 2, 4, 6, 11, 13, 14, 15 and 19) have the capacity to engage heat-shock protein chaperones as well as participate in R2TP-like complexes, thereby coupling the two chaperone activities. Based on the early onset of their gene expression during airway cell differentiation, DNAAF2, DNAAF5 and DNAAF13 are proposed to function within an early pre-assembly complex (Horani et al., 2018). Although DNAAF2 and DNAAF13 can bind HSP70 and HSP90 proteins, given their strong association with the R2TP complex, they could act as recruitment factors to bring chaperone-bound dynein arm client subunits to the R2TP ‘assembly platform’ for stable assembly.

DNAAF5 might act as an accessory to the R2TP complex, similar to the TTI proteins involved in the assembly and stabilisation of PIKKs such as mTORC1. DNAAF10 bridges the chaperoning activities of the R2TP and Prefoldin complexes. DNAAF17 could act as an accessory to R2TP via its interaction with DNAAF2. DNAAF19 binds microtubules *in vitro*, appears to form arrays and colocalises with cytoplasmic microtubules in cells (Falkenberg et al., 2021; King and Patel-King, 2015). Although it has been proposed to interact with the central pair microtubule-binding protein SPAG6, the RPAP domain of DNAAF19 strongly indicates an additional role as part of an R2TP-like complex.

DNAAF9, DNAAF12 and DNAAF18 have well-defined transport-related roles. DNAAF9 inhibits ODAs and additionally promotes their stable attachment to IFT trains (via IFT74 and IFT81) in human multiciliated cells (Issa et al., 2025). DNAAF12 associates with IFT (via IFT88) and is involved in the transport of the distal docking complex in *T. brucei* (Bonnefoy et al., 2018). DNAAF18 in *Chlamydomonas* was originally defined as a transport adaptor needed for the ciliary import of ODAs on IFT trains (via IFT46). However, the interaction between human DNAAF18 and IFT46 is not conserved (Wang et al., 2020). This indicates that there are species-specific differences in ODA transport mechanisms.

Intriguingly, both DNAAF9 and DNAAF18 have recently been defined as novel effectors of the ciliary small GTPase ARL3, which has well-established roles in ciliary cargo trafficking and release (Huang et al., 2024; Issa et al., 2025; Wang et al., 2025). Since DNAAF9 and DNAAF18 have both been linked to ODA transport (via IFT) as well as ARL3, there is likely to be a complex interplay between these factors and the ciliary import and subsequent regulated ciliary activation of axonemal dynein arms that needs further investigation.

## Outlook and future perspectives

Considering the scale of dynein arm assembly in a cellular context, it will be important to fully elucidate the crosstalk between the dynein assembly foci, within which DNAAFs are thought to operate, and different subcellular organelles such as the endoplasmic reticulum and mitochondria. The precise nature and role of these putative membraneless organelles remain open questions for future investigation. Specifically, elucidating the cellular sites of dynein arm assembly will require the application of advanced bioimaging methods such as expansion microscopy, correlative light and electron microscopy, and cryogenic electron tomography (cryo-ET).

Understanding the folding kinetics of individual dynein subunits, particularly the very large heavy chains, and the emerging enzymatic activities of the DNAAFs will require sophisticated biochemical and biophysical approaches. Future studies should focus on investigating the behaviour of partially folded/assembled intermediate states and the role of post-translational modifications in stabilising these folding and assembly intermediates. Cellular studies using more refined genetic perturbations such as CRISPR-mediated knock-in of pathogenic variants that perturb DNAAF interactions or functions within the dynein assembly pathway might also help pinpoint the steps at which assembly gets blocked, leading to the accumulation of misassembled intermediates in specific subcellular compartments.

Mechanistic studies on R2TP-like (e.g. R2SD, R2SP) co-chaperone complexes and their coordination with Prefoldin complexes should be a particular focus. The role of paralogous DNAAFs in R2TP-like complexes, and whether functional redundancy exists amongst them, remains an open question. For instance, RPAP3 is a paralogue of DNAAF13 and DNAAF19. Several species have only one RPAP domain-containing protein. RPAP3 might thus be involved in dynein arm assembly in species lacking DNAAF13 (SPAG1), in addition to its broader role in the assembly of other cellular machineries as part of the R2TP complex. Similarly, several species contain a variable number of PIH domain-containing proteins, indicating potential functional redundancy, unlike humans, which have four unique PIH proteins.

Further work is also needed to fully resolve the coupling mechanism between packaged ODAs and IFT trains across different species, as there are clear species-specific differences. For instance, whereas DNAAF18 is essential for coupling ODAs to IFT by directly interacting with IFT46 in *Chlamydomonas*, lack of this interaction in humans indicates that there are additional mechanisms by which ODAs could attach to IFT. Such differences might be a result of the diversity of ciliary structures that are evident across the tree of life. Additionally, there might be species-specific adaptations where ODA and IFT components have evolved to perform unique roles under specific contexts, such as to match the rate of ciliary assembly, which varies considerably across different species (for example, *Chlamydomonas* and *Tetrahymena* can assemble their cilia within hours, but human multiciliated cells can take several hours to days to build their motile cilia).

IDA transport into cilia is poorly understood and needs further investigation. The IDA3 protein in *Chlamydomonas* could be a potential IDA transport factor, but it does not appear to be conserved outside *Chlamydomonas* (Hunter et al., 2018; Wang et al., 2025). Similarly, Q22MS1 is a *Tetrahymena*-specific NDK domain-containing protein that could be involved in assembly and/or transport (Mali et al., 2021). There are likely to be several other species-specific adaptations that need to be investigated. Studies on dynein arm transport to cilia should also consider the role of a newly identified player – the ciliary small GTPase ARL3 – and elucidate how it coordinates dynein arm transport with their controlled release and activation specifically within the ciliary compartment.

Although numerous open questions related to the integrated molecular mechanisms of DNAAFs remain, recent insights have put a bright spotlight on this topic. Structure-guided mechanistic studies using cryo-EM and/or cryo-ET, new bioimaging methods such as expansion microscopy, and sophisticated biophysical and biochemical approaches, combined with innovative use of genetic model organisms, are likely to accelerate the pace of discovery and shed further light on the inner workings of the DNAAFs.

## Supplementary Material

10.1242/joces.264247_sup1 Supplementary information

Table S2. Accession numbers for DNAAFs from different species shown in Fig. 1.
