# Supplementary material for: Structural diversity and unity amongst axonemal dynein assembly factors
Source: J Cell Sci. 2025 Oct 28;138(20):jcs264247. doi: 10.1242/jcs.264247 (PMC12633730; doi:10.1242/jcs.264247)
Supplement: Supplementary information [file joces-138-264247-s1.pdf]

Table S1. Conservation of DNAAFs in commonly used ciliate model organisms.

Species / DNAAF	*Homo sapiens*		*Mus musculus*		*Danio rerio*		*Xenopus laevis*		*Drosophila melanogaster*		*Tetrahymena thermophila*		*Trypanosoma brucei*		*Caenorhabditis elegans*		*Chlamydomonas reinhardtii*	
	Uniprot ID	%ID	Uniprot ID	%ID	Uniprot ID	%ID	Uniprot ID	%ID	Uniprot ID	%ID	Uniprot ID	%ID	Uniprot ID	%ID	Uniprot ID	%ID	Uniprot ID	%ID
DNAAF1	Q8NEP3	100	Q9D2H9	77	Q7ZV84	65	A0A1L8GEJ5	46	Q8INT5	52	Q22BD9	50	Q382D9	36	x	x	Q09JZ4	43
DNAAF2	Q9NVR5	100	Q8BPI1	64	Q499A3	41	B1H1W9	52	Q0E9G3	43	I7M0T7	27	Q388W0	24	x	x	B5BUZ8	26
DNAAF3	Q8N9W5	100	Q3UYV8	74	F1Q7Z7	36	Q32NQ7	43	A1ZB91	31	Q24CJ1	34	Q387D1	26	x	x	F5A894	31
DNAAF4	Q8WXU2	100	Q8R368	79	Q6P010	54	Q6AZN0	63	Q9VKJ5	27	I7MDJ4	26	Q381I1	23	x	x	A0A286T4S6[$]	32
DNAAF5	Q86Y56	100	B9EJR8	78	A0A8M3AVC6	48	A0JMW2	55	Q8INF7	27	W7X7L3	23	Q585B8	23	x	x	A0A2K3DD76	31
DNAAF6	Q9NQM4	100	Q3KNI6	62	Q6IVV7	46	A0A1L8F6S4	44	Q9VUG3	31	Q23NK0	31	Q580K1	26	x	x	A0A2K3DK73	34
DNAAF7	O75800	100	Q99ML0	89	F1QN74	56	Q5FWU8	61	Q9VU41	31	W7X4E2	37	Q384J6	32	x	x	A0A2K3DGB3	32
DNAAF8	Q8IYS4	100	Q8C5G4	48	A0A8M2BBI0	26	A0A1L8EYB2	50	x	x	x	x	x	x	x	x	x	x
DNAAF9	Q5TEA3	100	Q7TT23	85	B8JIN4	53	A0A1L8HKG2	66	x	x	Q22YU3/Shulin	25	x	x	x	x	A0A2K3D7A4	26
DNAAF10	Q96MX6	100	Q8BGF3	94	Q561Y0	84	Q5M7F6	84	Q9VVM7	56	I7MG93	52	Q387Q4	44	x	x	A8J3F6	58
DNAAF11	Q86X45	100	O88978	79	B3DH20	54	A0A1L8G016	60	Q9VR52	43	Q233Z2	41	Q9NJE9	37	x	x	A0A2K3CRM8	42
DNAAF12	Q8IYG6	100	Q8K375	67	A0A8M3APS4	41	A0A1L8GJM2	46	Q9VW77	33	Q23KK9	32	Q387Y5	33	x	x	A0A2K3E7K0	30
DNAAF13	Q07617	100	Q80ZX8	69	F1RBN2	51	A0A8J0T515	48	Q9VBA1	32	I7MAP4/I7MAU8*	30	Q585V5*	34	Q18405*	30	A0A2K3D5T6	38
DNAAF14	Q9NWS0	100	Q9CQJ2	80	Q1RM55	40	Q7ZWY2	46	Q9VK57	28	I7MEI0	27	Q38DW7	34	Q19965	29	A0A2K3E4A1*	28
DNAAF15	Q8WWB5	100	Q8CHR9	72	Q5PRB3	40	A0A1L8FLS6	45	Q9VSY1*	30	I7M632	45	Q387R3	24	x	x	A0A2K3DCB0*	28
DNAAF16	P57076	100	Q8BL95	92	Q6DRC3	73	A0A1L8HCK2	73	Q9VZH1	50	I7LVP0	39	Q383T3	37	x	x	A0A2K3CU42	44
DNAAF17	Q9BRQ4	100	Q8CC70	84	I3ITQ2	50	Q5PQ82	65	x	x	I7M8T2	32	Q57Y38	21	x	x	A8IYS6	36
DNAAF18	Q8N136	100	D3Z7A5	90	Q1LV15	73	Q5FWQ6	80	Q9VAK0	38	I7MMK9	60	Q57W14	60	x	x	Q3Y8L7	62
DNAAF19	Q8IW40	100	Q9D9P2	81	Q6DGB6	50	A0A974BWN8	50	D5SHJ5*	46	I7MKE6	25	x	x	x	x	Q94EY1	31

This table shows conservation analysis of all 19 human DNAAFs across ciliated model organisms commonly used in cilia research. Uniprot ID and percentage sequence identity scores (%ID; coloured from high to low) for each assembly factor are shown for the different organisms. An ‘x’ denotes that no orthologue was detected by protein BLAST in a particular species. Most DNAAFs are missing in *C. elegans* as it lacks motile cilia; the two detected DNAAFs are part of the ubiquitous R2TP complex. DNAAF8 is only found in vertebrates. Ambiguous orthologue matches are denoted by asterisks. Such matches are likely due to the presence of paralogous proteins. The ‘$’ symbol denotes one of multiple matches that were detected: three DNAAF4-like proteins were detected in *C. reinhardtii* but only one is shown.

Journal of Cell Science • Supplementary information

Table S2. Accession numbers for DNAAFs from different species shown in Fig. 1.

Available for download at
https://journals.biologists.com/jcs/article-lookup/doi/10.1242/jcs.264247#supplementary-data

Journal of Cell Science • Supplementary information
